# Supplementary material for: Predicting visual function by interpreting a neuronal wiring diagram
Source: Nature. 2024 Oct 2;634(8032):113–23. doi: 10.1038/s41586-024-07953-5 (PMC11446822; doi:10.1038/s41586-024-07953-5)
Supplement: Supplementary file 6 — CRF and ERF predictions for individual TmY4 and TmY9 cells. Analogous to Supplementary Data 3, but for TmY target types. Shown are the top four monosynaptic pathways, the strongest pathway passing through each of the top ten intermediary types (ranking from Extended Data Fig. 7), and the trisynaptic pathway Tm1–TmY–Dm3–TmY (see the section entitled Prediction of spatial normalization). [file 41586_2024_7953_MOESM6_ESM.zip › DataS4/TmY9q/720575940631110487.pdf]

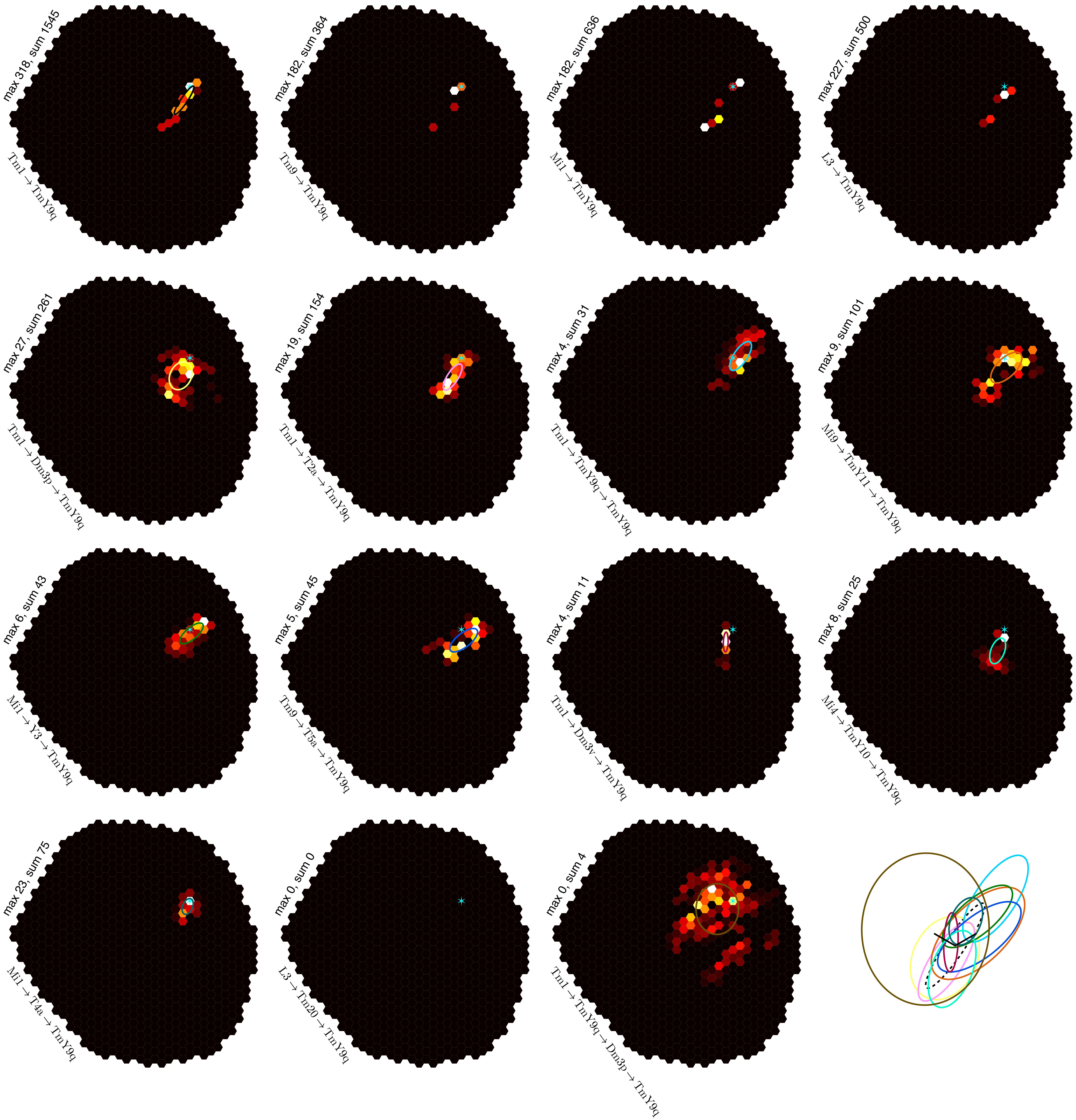
max 318, sum 1545
Tm1 → TmY9q
max 182, sum 364
Tm9 → TmY9q
max 182, sum 636
Mi1 → TmY9q
max 227, sum 500
L3 → TmY9q
max 27, sum 261
Tm1 → Dm3p → TmY9q
max 19, sum 154
Tm1 → T2a → TmY9q
max 4, sum 31
Tm1 → TmY9q → TmY9q
max 9, sum 101
Mi9 → TmY11 → TmY9q
max 6, sum 43
Mi1 → Y3 → TmY9q
max 5, sum 45
Tm9 → T5a → TmY9q
max 4, sum 11
Tm1 → Dm3v → TmY9q
max 8, sum 25
Mi4 → TmY10 → TmY9q
max 23, sum 75
Mi1 → T4a → TmY9q
max 0, sum 0
L3 → Tm20 → TmY9q
max 0, sum 4
Tm1 → TmY9q → Dm3p → TmY9q
